# The case for establishing a blockchain research and development program at an academic medical center

**DOI:** 10.30953/bhty.v4.161

**Published:** 2021-03-09

**Authors:** Muhammad Usman, Verena Kallhoff, Anjum Khurshid

**Affiliations:** 1 Department of Electrical and Computer Engineering, University of Texas at Austin, Austin, TX, USA; 2 Dell Medical School, University of Texas at Austin, Austin, TX, USA

**Keywords:** blockchain, health, academic medical center, research lab, market strategy

## Abstract

**Objective:**

To develop a research and development program to study factors that will support research, education and innovation using blockchain technology for health in an effective and sustainable manner. We proposed to conduct qualitative research to generate insights for developing a market strategy to build a research lab for the promotion of blockchain technologies in health in academic environments. The team aimed to identify the key barriers and opportunities for developing a sustainable research lab that generates research, education, and application of blockchain in healthcare at an academic medical institution and test those strategies in a real-world scenario.

**Methods:**

The research team identified potential customers and stakeholders through interviews and snowball sampling. The team conducted semi-structured interviews with 4 faculty researchers, 10 industry leaders, and 6 students from a variety of disciplines and organizations. The findings of these research activities informed our understanding of the needs of stratified customers and helped identify key assets and activities the lab will have to offer to meet those needs.

**Results:**

The research insights from data analysis were used to build the business model for establishing a blockchain in health impact lab. This systematic study of areas where blockchain technology can impact health will guide the future development of research agenda for the researchers on campus.

**Conclusion:**

Based on our learnings, we hope to design a Blockchain in Health Impact Lab to serve as a platform for students and faculty to come together with industry partners and explore current challenges of blockchain in healthcare. The academic medical center’s partnership with other healthcare providers will help create real-world opportunities to demonstrate and implement new technologies.

The COVID-19 crisis has created new challenges and underscored old ones, including the need for better information management systems. In healthcare, the pandemic has amplified the need for secure, timely, and accessible healthcare data—both for better patient care and effective public health policy. Blockchain promises to transform the way people interact with the health system—and their own health data. Handled intelligently, blockchain can make healthcare safer, accessible, secure, and inclusive (1–7). The technology is particularly well suited for emergencies like COVID-19 because it allows us to share information while preserving individual privacy (8–12). Blockchain-based information management offers efficient access to patient information to allow more informed treatment decisions, better collaboration between providers, and accelerated insights into emerging pandemics (13–15). But while we have identified myriad uses for blockchain in healthcare, there is still much to explore surrounding applications, logistics, ethics, and more.

Fulfilling the highest potential of this transformative technology requires collaborative, impartial leadership—a think tank with comprehensive resources. Deciding where such a think tank resides is of utmost importance. As one of the nation’s technology hubs, Austin is home to both startups and tech giants working in blockchain (16). It is also home to one of the nation’s great research institutions, The University of Texas at Austin, and the first new medical school at a top-tier university in half a century—Dell Medical School (DMS). Along with the school’s commitment to innovation in healthcare and value-based care, this convergence of resources positions DMS as an ideal partner in exploring how blockchain technology can revolutionize healthcare. The school has already shown leadership in using blockchain through MyPass, a joint initiative with the City of Austin and Austin-Travis County Emergency Medical Services (EMS) to give people experiencing homelessness secure, durable access to their health information (17).

Anecdotally, the team had received insights from leaders in healthcare that the high risks involved in testing new technology, combined with the lack of reviews and case studies from impartial organizations, lead to reluctance of testing blockchain solutions in the clinical setting, especially involving patient data. This prompted the team to set out to determine if and how a collaborative lab located at an academic medical center could serve as a convener and subject matter expert to identify opportunities, conduct research, and test solutions in a collaborative environment. We developed a research and development program to study factors that will support research, education, and innovation using blockchain technology for health in an effective and sustainable manner. The research focused on qualitative methods to collect data that helped identify key barriers and opportunities for developing a sustainable research lab that generates research, education, and application of blockchain in healthcare at an academic medical institution and tests those strategies in a real-world scenario.

## Research on blockchain in health at the University of Texas at Austin

Blockchain technology is being used in a variety of industries but its use in healthcare is limited. The COVID-19 crisis has made the need for better technology in health evident (15), yet even universities, including academic medical centers, are barely engaged in research on blockchain in health. DMS at the University of Texas at Austin was involved in the launch of the MyPass initiative led by the City of Austin. As part of this initiative, a digital wallet, containing social security number and other important records, is created for individuals experiencing homelessness. Upon validation by a notary, typically a social worker, service providers can access the information required for homelessness services (17). Other projects include a demonstration project at DMS for patient identity management called MediLinker and an analysis of peer-to-peer systems in terms of network scalability done at the University of Texas at Austin. DMS also partnered with the Austin Blockchain Collective to form a Health WorkGroup to promote academia–industry partnership (18). Austin Blockchain Collective is a group of over 100 companies hoping to make Austin a global hub for blockchain. The Collective seeks to bridge the gap between academia and industry partners with collaborators like the City of Austin and the University of Texas, as well as provide education on blockchain technology (19).

Current research on the use of blockchain technology in health at the University mainly relies on individual faculty or student initiatives and remains disjointed. Due to the multidisciplinary nature of this research and the potential impact of this technology on the multi-trillion-dollar health industry, it seems imperative that the community of researchers and students interested in this area have a place to coordinate and promote educational, research, and development activities focused on these new technologies in health. This led to the exploration of how best to set up a Blockchain in Health Impact Lab (BHIL) on campus.

## Methodology

### Our multidisciplinary team

The planning and management of this research was a collaboration between the Texas Health CoLab and the Division of Health Information and Data Analytic Sciences (HIDAS) at DMS with additional participants from a wide variety of colleges, schools, and departments across DMS and UT Austin. As a part of this study, we also engaged two UT Austin students. These students conducted literature reviews on previous blockchain-related publications at UT Austin and researched on blockchain hubs around the globe.

### Customer interviews

The research team identified potential customers and stakeholders through interviews and snowball sampling. The team interviewed 4 faculty members, 10 industry leaders, and 6 students from a variety of disciplines and organizations. The findings of these research activities were documented to build our understanding of the needs of stratified customers and to identify key assets and activities a blockchain lab will have to offer to meet those needs. The research insights from data analysis were used to build the business model set forth below. This systematic study of areas where blockchain technology can impact health will guide future development of research agenda for the researchers on campus. In general, we asked five research questions from our customers.

- **RQ1:** What are the gaps in the research and development of blockchain technology applications in healthcare?
- **RQ2:** Which specific problems can blockchain solve in healthcare?
- **RQ3:** Who are our customers? What are their needs?
- **RQ4:** How do we provide value? What products do we provide to satisfy our customers’ needs?
- **RQ5:** What will BHIL look like? How are we different?

The team used qualitative research methods to gain a deep understanding of the potential customers and their needs. Analysis of the interview responses and the learnings from lectures were used to develop a business model for the lab. The business model addresses the most relevant areas of blockchain research identified by stakeholders, outreach, and engagement strategy for researchers and industry partners in an academic medical center setting. The business model is a generalizable research product which serves as a template for other university-based groups to include a draft charter and marketing materials.

At the conclusion of the stakeholder interviews, the team of researchers reviewed the recorded conversations and assembled an in-depth understanding of the responses. Using an adapted version of the Business Model Canvas (20), the team built an understanding of potential ‘customers’ and their needs, the value proposition of a blockchain lab at a premier academic medical and research institution, key activities, key resources, key partners, and stakeholders. The team also developed a feasible cost structure for a sustainable resource providing unbiased insights and support for the use of blockchain technology in healthcare.

## Results

Below we report our findings from semi-structured interviews in the form of opinions expressed by our study participants. We also identify sources where some of these opinions have been expressed by others in published literature.

### RQ1: What are the gaps in the research and development of blockchain technology applications in healthcare?

Blockchain is a promising technology for healthcare but to contribute effectively to its widespread adoption or meaningful evaluation we started off by identifying the current concerns, doubts, and unknowns about this technology in health research and practice. Based on our key informant interviews, this section explores the gaps in the research and development of blockchain technology applications in health.

### Blockchain applications are at an infancy

Blockchain is a complex technology and most medical professionals lack technical knowledge about blockchain (21). They generally consider blockchain to be related to cryptocurrency. Due to this, they do not consider other potential applications of blockchain. There is a need to spread awareness of the ability of blockchain to solve some technological limitations in healthcare’s data and technology. Another challenge is that blockchain technology is relatively new and developers need more time developing solutions, while giving less time to User Interface/User Experience (UI/UX) development for end-user satisfaction. Consumers prefer applications with better usability designs, and current UI/UX for blockchain applications is not of a high standard (22).

#### Limited Blockchain R&D

Another challenge in the adoption of blockchain applications in health is the type of applications that can be developed using blockchain. Certain applications are suitable for blockchain, while for others, blockchain is not necessarily the best option (23). It is important to differentiate between these two sets of applications. However, in its early days, developers and researchers have tried to advertise blockchain technology for every application, even when it was not the most suitable approach. The result is customer distrust. Blockchain research is still limited in academia. Only a limited number of universities are doing research on blockchain, and even those universities are working on problems which do not directly benefit the industry. Big hospitals are buying smaller hospitals, and this is increasing interoperability issues (24). In some cases, one hospital chain can simultaneously have several different Electronic Medical Records (EMRs), legacy systems from smaller hospitals that merged, a physician organization, and potentially specialty clinics. The burden caused by the lack of interoperability is great, yet hospital leadership is weary of the uncertainty surrounding the implementation of a novel system and instead prefer to undergo the equally pain-staking process of migrating all systems to one EMR, if possible, because of more predictable outcomes.

#### Legal and regulatory issues

There are a lot of regulatory issues in the health industry. Medical data are highly confidential, and legal concerns need to be addressed before progress can be made in blockchain for health (25). Because of regulatory constraints, healthcare providers are hesitant to relinquish ownership of health data and insist on centralized authority over patient data rather than try a decentralized solution. Blockchain is not meant to store large amounts of data, yet many healthcare data files, such as imaging outputs, are very large. Also, lots of blockchain networks are coming into the market, and without industry standardization, it is possible and likely that blockchain interoperability issues will arise in the future. Having seen the challenges of fragmentation with the electronic medical record systems, which the industry is still struggling to solve, there is an understandable reluctance to adopt another technology that may have the same issues of proprietary platforms.

### RQ2: Which specific problems can blockchain solve in healthcare?

Blockchain is a suitable tool to address only specific problems, and identifying these problems in healthcare is an important task to accomplish. Our interviews with experts elucidated some of the problems in health that can be solved using blockchain technology.

#### Supply chain management

Blockchain is commonly used in supply chain logistics outside of healthcare already allowing for an immutable tracking of items along the path from production to end consumer (26–30). Likewise, blockchain could keep track of medical samples, vaccinations, and medicines. A key-informant, a pathologist, emphasized that a unified way to track tissue samples and associated diagnoses would be beneficial to reference labs. In addition, clinics, transportation companies, and labs could benefit from interoperable software.

#### Tracking credentials/licenses

Credentialing of doctors or nurses currently takes many months and is a highly manual task (31, 32). If the education and practice history of physicians were on the blockchain, providers could verify that a health professional holds valid credentials prior to hiring, eliminating delays and frustration currently experienced. Such credentialing is very similar to the use of educational degrees and diplomas to be verified by academic institutions.

#### Tracking personal medical data

Genomic data from the tissue samples are not tracked, and therefore, patients cannot benefit when these data are used by pharmaceutical companies for research. If the record of each sample is stored on the blockchain, then patients will be able to control their data and benefit in monetary terms when a pharmaceutical company wants to use their data for research purposes. Similarly, the digital identity of people experiencing homelessness can be implemented using blockchain (33). Blockchain can also be used to store links to all the medical records of an individual, even if the records are stored in multiple different locations. The patients can also exercise more control over their medical records and their ability to track their own records (34).

#### Interoperability

Blockchain technology has been used to support Internet of Things (IoTs) allowing for identification and coordination among computers and electronic equipment. The Internet of Medical Things can also use the immutability and auditability features of blockchain as one of the possible solutions for interoperability (35).

#### Research

Blockchain can be used to store and catalog medical research so that every research work can be audited to confirm its validity (36–38). Research papers can be peer reviewed using blockchain leading to more visibility in research.

#### Other health applications

Blockchain will also be helpful when a patient changes his/her insurance (39). The previous insurance company can share the keys with the new insurance company. This will greatly improve interoperability. Blockchain can also be used for payroll management and medical billing (40). Prescriptions can be managed using blockchain (41). Pharmacies can verify that the prescriptions are valid and issued by an accredited doctor. Drug trials can be recorded on the blockchain to speed up the procedure for FDA approval (42). Health insurance settlements can take weeks or even months to complete with the current technology. However, with blockchain, it can be achieved within a day (39).

### RQ3: Who are our customers? What are their needs?

Based on the interviews conducted between April 2020 and July 2020, it became clear that there are diverse groups who could benefit from engaging with an academic consortium focused on blockchain technology and based at an academic medical center. Of course, all interviewees mentioned the complexity of the health industry with its broad range of providers, practitioners, payers, and other stakeholders. These groups are interested in more efficient and secure tools to improve their enterprises, yet they are also wary of novel technologies and the validity of the promises its proponents make. Hence, access to unbiased information and education on the realistic goals of blockchain technology in health are of utmost importance.

Another group that was regularly mentioned was the technology industry. Also consisting of a wide variety of stakeholders, their primary interests are opportunities to test and develop better tools as well as access to key opinion leaders and opportunities to network and learn from leading voices in the industry. Networking with each other, research, and opportunities to engage their future workforce are also very high on the list for these stakeholders. As blockchain technology faces many hurdles in acceptance, industry is keen on developing a shared voice to provide unbiased information to potential users.

A third group frequently mentioned were legislators and regulators, as well as public health officials and other governmental bodies. These stakeholders are keenly aware of the challenges of the US health system and have been hearing promises from technologists for many years. Before enabling, recommending, or implementing sweeping changes, much work must be done to perform in-depth research to truly understand the diverse implications of enabling a new technology to provide tools with much broader functionality. These stakeholders are in dire need of unbiased information obtained through collaborative research, case studies that allow for a broader understanding of implications, and access to key opinion leaders that can provide insights and guidance. This group of clients is also in need of more efficient and secure tools, but they must come with the confidence that accompanies thorough unbiased testing. In addition, this group is an important stakeholder in that it can unlock and enable the use of blockchain technology through some of the required regulatory changes. Again, the confidence for regulatory changes will come through unbiased information and education that is best obtained through an academic research enterprise.

Another important external client to an academically based Blockchain in Health Impact Lab (BHIL) comprises the patients, patient advocates, and privacy groups. This group has the potential of gaining substantial independence and improvement in their interaction with the health industry by using blockchain technology. Access to personalized medicine, the ability to set permissions for use of their personal health information, promises more coordinated and personalized care while decreasing risks for medical errors and omissions due to incomplete or missing data (34, 43). Yet, patients, patient advocates, and privacy groups are also rightfully concerned that sweeping changes in underlying technologies may impact the privacy and security of health data, benefitting only the larger corporations with little recourse and opportunity for them (44). As in prior instances, access to unbiased education and information that feeds into regulation and better-informed public opinion is crucial to gain the trust and support from this group of clients and enable them to reap the benefits of this technology.

A crucial internal client for the BHIL is the on-campus community comprising faculty and student researchers. Eager to understand the strengths and weaknesses of the technology in the health setting more deeply, this group is looking for opportunities to test and develop better technological tools, build case studies, and engage in research around deeper implications of the technology. The ability to engage with other professionals in this field provides opportunities to understand the broader implications of their research work, thus enabling more refined case studies and technologies. In addition, professional events, sponsored research projects, and educational opportunities allow trainees to network with potential future employers as well.

Some of the above observations and comments from experts are summarized in the Table 1.

**Table 1:** Stakeholder needs for blockchain in health research

Customer	Needs
Health Systems, Providers and Payers	Better, more efficient, and secure tools; access to unbiased information and education
Industry	Opportunities to test and develop better tools; access to key opinion leaders; access to ideas, collaboration to develop better, more efficient, and secure tools; future workforce
Legislators, Government, Regulators, Public Health Officials	Better, more efficient, and secure tools; access to unbiased information and education
Patients, Patient Advocates, Privacy Groups	Better, more efficient, and personalized care and access; access to unbiased information and education
Faculty and Student Researchers	Opportunities to research, test, and develop better tools; education; future career opportunities

### RQ4: How do we provide value? What products do we provide to satisfy our customers’ needs?

The major needs that we identified from the key informant interviews were applied research, new tools, education, and an unbiased source of information on blockchain technology.

#### Research and development

Different research groups are working on various health blockchain projects. The results are published in various conferences and symposiums (45). However, there is no unified repository which combines all the health blockchain-related research. BHIL can help create such a repository and determine what are the lessons learned from each project. There is a need to engage with politicians and decision-makers to shape public policy because new rules need to be made so that pharmacies and healthcare providers can be encouraged to implement well-tested and impactful blockchain solutions.

#### Awareness campaign and research projects

BHIL can start an awareness campaign to educate people and funding organizations about the advantages of blockchain. There is also a misunderstanding that one blockchain framework can be used to support all types of applications (23). The lab can help to educate about different blockchain platforms that may be required to implement different applications. Blockchain in health is assumed to solve the interoperability problem. However, a detailed study needs to be done to see if blockchain systems of two different companies can work together. The academic knowledge needs to be published, and the lab can arrange regular conferences in collaboration with research experts. It can bridge the gap between industry and academia by asking companies for relevant projects which can be completed by students, and students in return can be financially supported. This way, students will work on projects which will be used in real settings. The lab can arrange happy hours between industry and researchers. The lab can also provide opportunities to companies to participate in academic and translational research in blockchain.

#### Research conferences and consortiums

BHIL can invite panels to discuss current market developments in blockchain. BHIL can also bridge the gap between stakeholders by including research groups and companies from the Austin Blockchain Collective. BHIL can bring technology companies, researchers, students, and healthcare providers to the table and discuss blockchain use cases in health. It can act as a platform for experts interested in blockchain applications and technology.

Given the feedback from our interviewees, DMS is launching the BHIL as a collaboration between the school’s Division of HIDAS and its product innovation initiative, the Texas Health CoLab. The BHIL will serve as a platform for educational seminars and panel discussions addressing clinical, industry, and community-based stakeholders. It will highlight the power of technology to solve some of our most pressing problems and gather experts for workshops to explore challenges, solutions, ethical concerns, regulations, and policies. BHIL will endeavor to become a preeminent resource for unbiased information on the use of blockchain technology in healthcare. Further, BHIL will bring together students and faculty in a variety of disciplines to come together with industry partners to explore current challenges of blockchain in healthcare, identify use cases, develop, implement, test, validate, and partner to disseminate promising solutions.

The school’s partnership with other healthcare providers will help create real-world opportunities to demonstrate and implement new technologies. BHIL will become a resource and partner for both public and private organizations as the blockchain community advances this emerging field and develops future leaders in the field of blockchain in health. The lab will provide access to experts in ethics, legal, computer science, clinical care, information sciences, finance, and more from all over campus as well as a platform to build and test new applications in health.

BHIL is envisioned to be an unbiased and collaborative platform to advance the use of blockchain technology for the public good. It is essential that the core activities of the lab have impartial funding. Hence, the leadership team will be working to obtain funds from philanthropic sources or through local and federal grants. While open to collaborating with specific partners on projects in areas such as research, innovation, problem definition, or commercialization of particular interest to the partner, the essence of BHIL is to become a preeminent, trusted resource for all things blockchain and healthcare.

The research team analyzed the key resources available to further the mission to become a lab created to bridge the gap between academia and industry, different industry sectors and research groups, relying on UT Austin’s immense research faculty with diverse research backgrounds and its talented students. The DMS is working with faculty from the College of Computer Science, the iSchool, McCombs Business School, the LBJ School of Public Affairs, and many others. Promoting and supporting the Vision of UT Austin: ‘What starts here changes the world’ is embedded in the work of the BHIL. Table 2 summarizes some of our findings in this section.

**Table 2:** Blockchain in Health Impact Lab (BHIL)’s value propositions

Need	BHIL value proposition
Better, more efficient, more secure tools	Generate impactful and applied research, case studies, accelerate out of the lab into the marketplace
Education	General, free access to monthly lectures; curriculum for healthcare providers, healthcare leaders, and others; technology curriculum (there are several tools out there already), seminars, workshops
Access to knowledge and unbiased information	Generate research, peer-reviewed publications, case studies
Testing ground and development of better tools	Opportunities for sponsored research—through standardized agreement for BHIL members
Opportunities to research, ideas for problems	Industry sponsored research, access to collaborators for large grants, painstorming, opportunities for networking and hearing about research work
More efficient and personalized care and access	Generate impactful and applied research, case studies, accelerate out of the lab into the marketplace
Access to future workforce	Opportunities for networking and exchanging about work, sponsored projects, internships
Access to KOL, discussion platform	Seminars, workshops, summits and 1–2-day-long events

### RQ5: What will BHIL look like? How are we different?

**Fig. 1:**
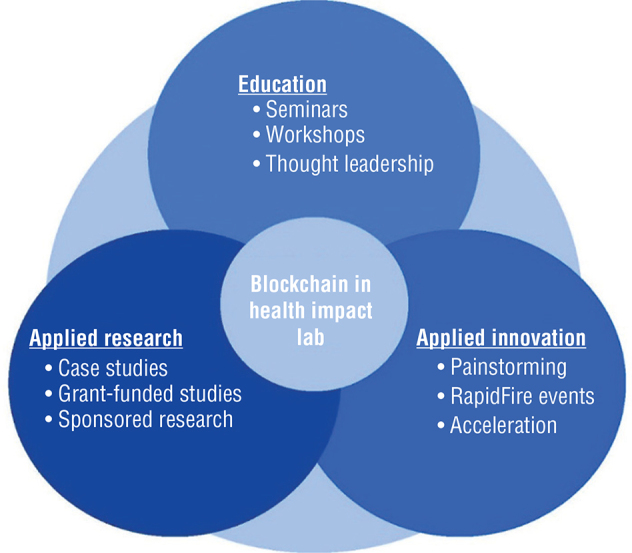
Key activities at BHIL.

The key activities of BHIL fall into three main categories: Applied Research, Applied Innovation, and Education (Fig. 1). Each category has distinct and major activities but as part of the collaborative BHIL, all integrate and overlap to ensure that projects and ideas can seamlessly move from one into the other. Work done during thought leadership conversations may lead to a case study led by the Applied Research team, and a promising project has the opportunity for a seamless hand-off to the Applied Innovation team for acceleration into a commercial product. At the same time, a problem identified by a diverse set of stakeholders can move from painstorming to applied research and subsequently be presented and discussed during a seminar. The setup of the BHIL as a collaboration between the applied research and the innovation initiatives at DMS is chosen deliberately to allow for a smooth transition and truly impactful research and innovation.

## Future work and conclusion

BHIL strategy for sustainability will start with a draft charter, marketing assets, and a draft pitch. In preparation for a possible Stage II, the team will identify additional meetings or conferences to meet with potential industry partners to the lab and evaluate the interest in affiliating with the lab. Potential partners may include local and global companies like Ripple, Cognitive Scale, Factom, IBM, Dell, Google, and others.

The BHIL will become a resource for both public and private organizations as we advance this emerging field and train tomorrow’s workforce. The lab will provide access to experts in ethics, legal, computer science, clinical care, information sciences, finance, and more from all over the campus. It will develop research studies and programs that identify, research, and address opportunities and challenges for the use of blockchain technology in healthcare. Research projects will address such topics as pandemic emergency needs, healthcare data flow and security, clinical research, pharmaceutical studies, identity management, and data analysis. The partnership will also develop programs that engage students, faculty, technologists, and medical providers to find challenges in healthcare, create solutions, and share knowledge about the use of blockchain in healthcare.

Blockchain opens the possibility of a whole new world in medical data management and the ability of individuals to control their own data. Decentralized, secure, easily accessible information can empower patients and transform healthcare, and we are just beginning to see blockchain’s vast potential. But there are challenges, and before we solve them, we must identify them. DMS seeks to explore how blockchain can—and should—revolutionize healthcare. The time for blockchain is now, and this is the place.
